# Statin Pharmacogenomics: Opportunities to Improve Patient Outcomes and Healthcare Costs with Genetic Testing

**DOI:** 10.3390/jpm2040158

**Published:** 2012-10-17

**Authors:** William J. Canestaro, David G. Brooks, Donald Chaplin, Niteesh K. Choudhry, Elizabeth Lawler, Lori Martell, Troyen Brennan, E. Robert Wassman

**Affiliations:** 1 Generation Health, 130 Turner St., Waltham, MA 02453, USA; 2 TESARO Inc., 1000 Winter St. Suite 3300, Waltham, MA 02451, USA; 3 Division of Pharmacoepidemiology and Pharmacoeconomics, Department of Medicine, Brigham and Women’s Hospital, 1620 Tremont St., Suite 3030, Boston, MA 02120, USA; 4 ZIOPHARM Oncology, Inc., 1 First Avenue, Parris Building, #34, Navy Yard Plaza, Boston, MA 02129, USA; 5 CVS Caremark, One CVS Drive, Woonsocket, RI 02895, USA

**Keywords:** statins, adherence, myopathy, *KIF6*, *SLCO1B1*

## Abstract

HMG-CoA reductase inhibitors, commonly known as statins, are some of the most widely prescribed medications worldwide and have been shown to be effective at lowering cholesterol in numerous long-term prospective trials, yet there are significant limitations to their use. First, patients receiving statin therapy have relatively low levels of medication adherence compared with other drug classes. Next, numerous statin formulations are available, each with its own unique safety and efficacy profile, and it may be unclear to prescribers which treatment is optimal for their patients. Finally, statins have class-wide side effects of myopathy and rhabdomyolysis that have resulted in a product recall and dosage limitations. Recent evidence suggests that two genomic markers, *KIF6* and *SLCO1B1*, may inform the therapy choice of patients initiating statins. Given the prevalence of statin usage, their potential health advantages and their overall cost to the healthcare system, there could be significant clinical benefit from creating personalized treatment regimens. Ultimately, if this approach is effective it may encourage higher adoption of generic statins when appropriate, promote adherence, lower rates of myopathy, and overall achieve higher value cardiovascular care. This paper will review the evidence for personalized prescribing of statins via *KIF6* and *SLCO1B1* and consider some of the implications for testing these markers as part of routine clinical care.

## 1. Introduction

Coronary heart disease (CHD) is a problem of epidemic proportions that is estimated to be responsible for more than 400,000 deaths annually in the United States (US) [1]. Furthermore, elevated levels of low density lipoprotein (LDL) cholesterol, one of the main risk factors for CHD, can be found in more than a quarter of all American adults [2]. Large scale randomized controlled trials have demonstrated the ability of HMG CoA (or 3-hydroxy-3-methyl-glutaryl-coenzyme A) reductase inhibitors, known as statins, to lower LDL levels and prevent major coronary events. As a result, nearly 20 million Americans regularly used a lipid lowering agent in 2011 resulting in greater than $20B in spending for the US healthcare system [3]. Although statins are generally regarded as having a mild side effect profile, they have been plagued by a class-wide side effect of muscle toxicity leading to both dosage limitations and recalls [4,5]. In addition, patients taking statins have relatively low levels of medication adherence [6,7,8]. Medication nonadherence across all drug classes is a serious public health concern. Despite evidence that high levels of medication adherence can improve clinical outcomes and quality of life [8,9,10], it has been estimated that up to half of the 3.2 billion prescriptions dispensed annually are not taken as prescribed [11]. This has adverse clinical and economic implications for the healthcare system. Estimates suggest that poor adherence across all drug classes may result in 33%–69% of all hospital admissions [11] and up to 125,000 deaths annually [12], resulting in $100–300 billion in avoidable medical spending annually [11,13,14,15,16].

The issue of nonadherence is especially relevant in the treatment of elevated cholesterol. Nonadherence to statin treatment has serious health consequences and has been associated with a 50% reduction in the survival benefit seen in trials [17] and increased annual medical spending of $1,860 per patient [18]. Although the benefits of long-term statin therapy and the critical role of adherence for the prevention of atherosclerosis and subsequent clinical events have been proven, adherence rates remain low. Among statin-treated patients, observational studies report 1-year discontinuation rates between 15% and 60% depending on the practice setting and patient population [6,7,8]. These discontinuation rates can approach 75% after 2 years in patients receiving statins for primary prevention of CHD [19]. 

Patients do not adhere to their medications for many reasons including low health literacy, cost, inability to feel the drug’s therapeutic effect, and side effect profiles [13]. In response to these barriers, new methods of patient education as well as other strategies such as adjusting the treatment duration, regimen, requirements for lifestyle change, and cost have been employed [11,13,20]. In general, these interventions have shown limited effectiveness and thus highlight the importance of novel strategies to promote adherence. More recently, investigators have also studied the impact of genetic risk disclosure to the patient as a tool to improve medication adherence with mixed results [21,22,23,24,25]. In the case of statins, *KIF6* and *SLCO1B1* have each been proposed as clinically-valid biomarkers to facilitate personalized statin treatment and improve adherence [26,27]. The evidence for utilizing each of these genes to personalize statin treatment is reviewed below.

## 2. *KIF6* as a Prognostic and Predictive Marker

The KIF6 protein is a member of the kinesin family, which is responsible for the intracellular transport of messenger ribonucleotides (mRNA’s), protein complexes, and organelles. Kinesins are dimeric molecules consisting of a ‘tail’ at the C-terminal domain that interact with the cellular cargo and a ‘head’ at the N-terminal domain that has the ability to move along microtubules in a mechanism that closely resembles bipedal locomotion [28]. The 2155T>C single nucleotide polymorphism (SNP) replaces a non-polar tryptophan residue with a polar arginine near the presumed binding domain for cellular cargo. Although the mechanism by which this variant exerts its phenotypic effects has yet to be defined, it is possible this amino acid substitution may alter the affinity of the binding domain for cargo proteins and or modify the kinesin’s motor activity [29].

Kinesin-like family 6 (*KIF6*) is potentially both a prognostic marker of coronary heart disease risk and a predictive marker of statin efficacy. Carriers of the *KIF6* 2155T>C allele display a greater risk for coronary events as well as greater benefit from statin therapy [30]. As an adherence intervention, if *KIF6* testing were able to identify those patients with the greatest net benefit from statin therapy, it may improve a patient’s sense of self-efficacy [31] and subsequent adherence [32]. 

A polymorphism in *KIF6* has been associated both with risk of coronary heart disease [33,34] and response to statin treatment [35,36,37]. This *KIF6* 2155T>C variant (denoted rs20455 in SNP database) is relatively common and if validated clinically, could present a useful tool for identifying which patients would most benefit from statin therapy. Although this allele is relatively common, it does have significant variation in frequency across ethnic groups (Table 1).

**Table 1:** *KIF6* Genotypes in Various Ethnic Groups.

Nucleotide Change	rsID	Protein Variation	Allele Frequency(%) ^a^				Ref.
			AA	Hs	As	C	
**2155T>C**	20455	Trp719Arg	78	36	50	36	[29]

^a^ AA = African American; Hs = Hispanic; As = Asian (Chinese and Japanese); C = Caucasian.

### 2.1. Clinical Evidence

The initial evidence supporting an association between the 2155T>C SNP and either CHD or statin response consists of genetic association studies conducted retrospectively as *post hoc* analyses in large clinical trials [35,36,37,38,39]. These studies have recently been viewed with some skepticism, in part since they were published by authors closely affiliated with the sole commercial distributor of a *KIF6* test in the US, but more so due to the underlying methodology of a “candidate gene” approach instead of a more impartial “genome-wide association” study [40,41]. Furthermore, subsequent independent retrospective association studies failed to replicate the results found in the initial analyses [42,43,44]. Finally, two meta-analyses of 19 case-control studies of nonfatal CHD that did not assess the effect of statin treatment on disease progression failed to show the previously reported association between *KIF6* and disease progression [30,45]. 

The studies evaluating the utility of *KIF6* testing can roughly be broken into two categories: those assessing its utility as a predictive marker of a patient’s response to statin therapy, and those assessing its utility as a prognostic marker of disease progression. 

#### 2.1.1. Evidence of KIF6 and Statin Response

To date, eight retrospective genetic association studies have been conducted which tested the hypothesis that 2155T>C carriers experience a greater coronary risk reduction from statin therapy than non-carriers (Table 2). 

**Table 2:** *KIF6* and Statin Response.

Study	Arms ^a^	(n) ^b^	Primary Outcome	Hazard Ratio: More *vs.* Less/No Statin ^c^ (95% CI)		*p* value
				Non-carriers	Carriers	
**CARE **[39]	P, Pl	2,746	MI	**0.80 **(0.52–1.24)	**0.63** (0.46–0.87)	<0.005
**WOSCOPS **[39]	P, Pl	1,527	CHD	**0.91 ^d^** (0.64–1.28)	**0.50 ^d^** (0.38–0.68)	<0.005
**TIMI-22 **[37]	A 80 mg, P 40 mg	1,778	CHD	**0.94** (0.70–1.27)	**0.59** (0.45–0.77)	<0.005
**PROSPER **[36]	P, Pl	5,752	MI	**0.94** (0.69–1.28)	**0.66 **(0.52–0.86)	<0.005
**HPS **[42]	S 40 mg, Pl	18,348	Any Major Vascular Event	**0.76** (0.69–0.83)	**0.77 **(0.71–0.84)	NS ^e^
**TNT **[44]	A 10 mg, A 80 mg	4,599	Any Major Vascular Event	**0.81** (0.59–1.11)	**0.85** (0.66–1.11)	NS
**IDEAL **[44]	S 20–40 mg, A 80 mg	6,541	Any Major Vascular Event	**0.85** (0.67–1.10)	**0.88** (0.72–1.07)	NS
**JUPITER **[43]	R, Pl	8,781	Any Major Vascular Event	**0.57** (0.39–0.83)	**0.63** (0.47–0.84)	NS

^a^ P = Pravastatin; Pl = Placebo; A = Atorvastatin; R = Rosuvastatin; ^b^ Number included in genetic analysis; ^c^ Hazard ratio of defined endpoint between intervention and control arms; ^d^ reported as odds ratio, ^e^ Result not statistically significant (*p* > 0.05).

**Figure 1:**
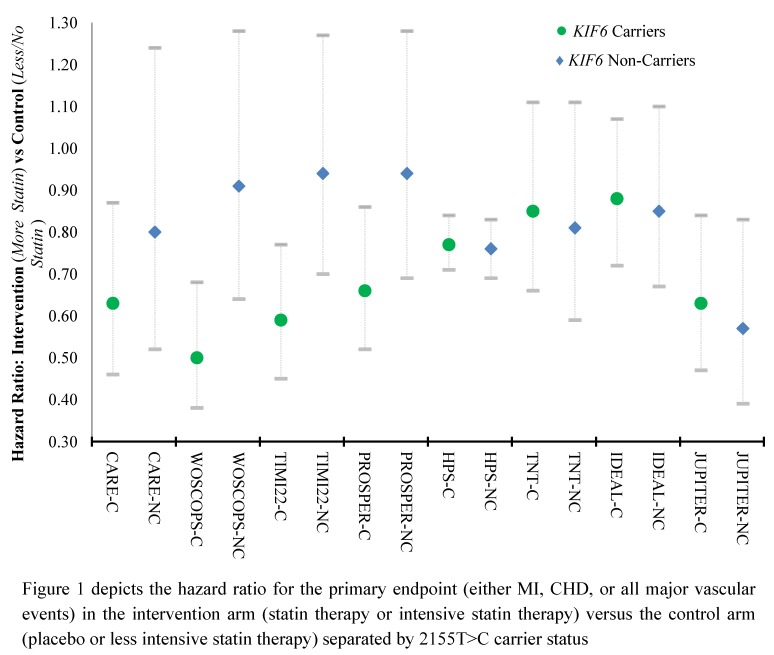
KIF6 and Statin Response.

The first four of these analyses conducted in the Cholesterol and Recurrent Events (CARE), West of Scotland Coronary Prevention Study (WOSCOPS), Thrombolysis in Myocardial Infarction-22 (TIMI-22), and Prospective Study of Pravastatin in the Elderly at Risk (PROSPER) cohorts indicated that while carriers of the 2155T>C SNP mutation experience a significantly lower coronary event risk, non-carriers show no statistically significant decrease in their baseline event rate with statin therapy [36,37,39]. Counter-intuitively, although carriers experienced a reduction in clinical events on therapy, they did not have a statistically significant difference in reductions of low-density lipoprotein (LDL) cholesterol or inflammatory markers during statin treatment [30]. This result implies that while carriers are selected for response to statin therapy, their improved outcomes are not derived from decreased levels of LDL. Although statins may have a pleiotropic effect beyond LDL reduction, this is still the primary target of therapy making this finding extremely controversial. Subsequent analyses in the Heart Protection Study (HPS), Treating to New Targets (TNT), Incremental Decrease in End Points Through Aggressive Lipid Lowering (IDEAL), and Justification for the Use of Statins in Primary Prevention: An Intervention Trial Evaluating Rosuvastatin (JUPITER) cohorts all failed to replicate the differences in clinical events in carriers and non-carriers observed in earlier trials (Figure 1) [42,43,44]. 

Importantly, there are some key structural differences between the earlier and more recent trials which may account for some of this difference in effect size. Namely, patients enrolled in the later trials had a lower LDL-C at the time of randomization. This could ameliorate some of the risk reduction from statin therapy and mask some of the differential effect of a potentially deleterious KIF6 mutation. Recently, a meta-regression analysis focused on this discordance concluded that *KIF6* may be responsible for mediating the deleterious of effects of LDL, thereby increasing a patient’s susceptibility to increased blood levels [30].

#### 2.1.2. Evidence of *KIF6* and Risk of Coronary Heart Disease

It is possible that the benefit of statin treatment among carriers found in earlier analyses may have nothing to do with the pharmacologic mechanism of their statin treatment but instead may be due to an independent risk of CHD progression. This hypothesis has also been evaluated in several analyses. Similar to the analyses evaluating the statin efficacy argument, this ‘prognostic risk’ hypothesis was supported by early investigations [33,34,38], yet failed to be replicated in later analyses. A large meta-analysis of 19 case control studies of >50,000 patients showed no statistically significant effect of carrier status on disease risk [45]. Again there are some caveats to this analysis as well. First, it did not include information on patients statin usage which is an important effect modifier of KIF6 carrier status. Next, this study primarily included case control studies of angiographically defined coronary artery disease (CAD) as opposed to CHD events that were the primary focus of the WHS, ARIC, WOSCOPS, CARE, and CHS studies.

### 2.2. AKROBATS and KIF6 as a Tool for Promoting Adherence

With traditional adherence-promoting interventions, much of the benefit comes from improving a patient’s self-efficacy or belief that their actions will have meaningful impact on their outcomes. Logically, if *KIF6* testing were able to identify those patients with the greatest benefit from statin therapy, it might also have utility as an adherence-promoting intervention via its ability to improve a patient’s sense of self-efficacy [32]. The potential utility of routine *KIF6* testing to promote adherence was evaluated in the Additional *KIF6* Risk Offers Better Adherence to Statins (AKROBATS) trial. AKROBATS was a non-randomized comparative effectiveness study where enrolled patients were offered *KIF6* testing, and their subsequent adherence to treatment was compared with concurrent untested controls via a prescription database [27]. This hypothesis-generating study indicated that patients who were aware of their *KIF6* status were approximately twice as likely to be adherent to therapy at 6 months based upon a proportion of days covered (PDC) value greater than 0.80 [46].

AKROBATS was limited by its non-randomized and relatively uncontrolled design. The portion of the improvement in adherence as due to the patient’s knowledge of their *KIF6* carrier status was difficult to determine. In fact, it is likely that much of the improvement that was seen was due to the fact that in participating in the trial, patients were in conversation with a pharmacist about their coronary risk and the need for good adherence. In itself, such a conversation is an adherence-promoting intervention independent of any genomic risk information that was disclosed. 

Although the evidence from the AKROBATS trial may not fully support the utility of *KIF6* testing, the concept is compelling. The trial presents an important proof-of-principle that interventions aimed at genomic personalization of statin therapy have the potential to improve adherence, and thereby patient outcomes of patients on statin therapy. Other markers, such as *SLCO1B1*, are now emerging as alternative pharmacogenomic markers that have clear evidence and biological plausibility and may operate in robust ways. 

## 3. *SLCO1B1* and Statin-Related Myopathy

### 3.1. Epidemiology

Although statins have well established efficacy in lowering atherosclerotic cardiovascular event and death rates and are generally regarded as safe drugs, muscle pain and weakness (myalgia/myopathy) are common side effects in this class, occurring in up to 10% of patients [47]. The concern over statin-related myopathy (SRM) is exemplified by the drug cerivastatin, which the FDA removed from the market due to its high risk of rhabdomyolysis and subsequent deaths [4]. Recently, the concern over SRM motivated the FDA to place new warnings on simvastatin formulations due to the significant risk of both myopathy and rhabdomyolysis with high doses [5,48]. 

SRM is an extremely heterogeneous condition. Patients with SRM can present with muscle complaints ranging from weakness, aches and/or pain without elevated creatine kinase (CK) levels, (*i.e*., myalgia), to more significant discomfort with mild CK elevations and myositis, to life threatening rhabdomyolysis [47]. Myalgia is the least severe but most common presentation of muscle toxicity, and rhabdomyolysis with potential renal failure is the most severe but least common presentation occurring in only a small percentage of the patients who develop myopathy. Although the exact rate of myopathy is unclear, outcomes data suggest that it may be more common than originally thought, based on strictly-controlled pre-approval clinical trials. Studies of intensive statin therapy report that roughly 3% and 2% of patients will experience myalgia and myopathy, respectively [49]. In studies that incorporate patient reported outcomes, however, the prevalence of muscle related side effects are as high as 10%–25% [50,51]. 

### 3.2. Myopathy and Adherence

Although SRM actually relatively infrequently leads to hospitalization or disability, its milder clinical effects are an important cause of statin intolerance and discontinuation [52,53]. Previous reports have repeatedly suggested that side effects or the perception of side effects may be among the most significant obstacles to optimal adherence on statin treatment, and a major source of random switching as well [54,55,56]. Thus, while myopathy may be dismissed by some prescribers as minor aches and pains, it may have a more profound consequence in terms of treatment discontinuation. The results from the USAGE study (Understanding Statin Use in America and Gaps in Patient Education) which was an internet-based survey of more than 10,000 statin users highlight this issue. Twenty-nine percent of all participants had experienced muscle-related side effects, and of those who had discontinued their medication due to a side effect, approximately one third did so without speaking with their physician [50]. In other words, some patients who would likely benefit from statins discontinued therapy outside of the healthcare system. The gravity of this problem may be invisible to many health care providers.

## 4. *SLCO1B1* as a Predictive Marker

Solute carrier organic anion transporter family member 1B1 (*SLCO1B1*) is a predictive marker of statin-related myopathy (SRM) which is a significant barrier to optimal adherence. Although there are many clinical factors that may predispose a patient to SRM, recent evidence suggests that SRM has a very strong genetic component. In fact, up to half of the SRM associated with simvastatin, one of the most commonly prescribed statins, may be attributable to a single genetic variant in *SLCO1B1* [57]. Furthermore, recent evidence has demonstrated that patients carrying certain variants in *SLCO1B1* are twice as likely to show signs of intolerance to the first statin they are prescribed, which can lead to trial-and-error prescribing and unnecessary drug churn [26]. These findings suggest that identification of patients with variant forms of *SLCO1B1* could mitigate SRM and subsequent low adherence.

### 4.1. SLCO1B1 Physiology

**Figure 2:**
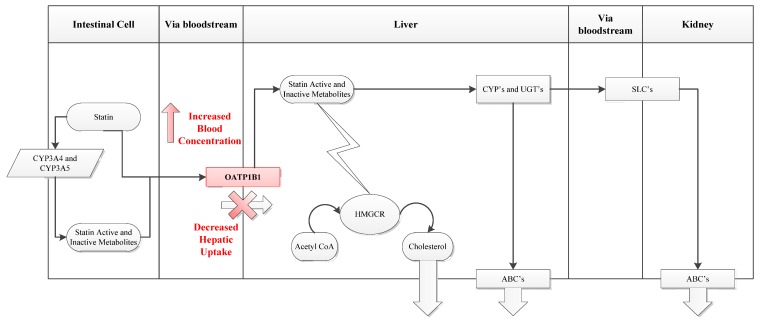
Statin Uptake Pathway. (**a**) *SLCO1B1* encodes the OATP1B1 influx transporter. (**b**) OATP1B1 transport is particularly important for hepatic accessibility of statins. The transporter contributes to liver uptake of statins including first pass clearance from the portal circulation so that decreased transport results in increases systemic (including muscle) exposure to statin. (**c**) HMGCR = 3-hydroxy-3-methyl-glutaryl-CoA reductase, CYP = cytochrome P450 isoenzymes, UGT = UDP-glucuronyl transferase class of enzymes, SLC = solute carrier group of membrane transporters, ABC = ATP-binding cassette transporters.

For statins to perform their function they must first reach the liver. The uptake of statins from portal blood into hepatocytes across the phospholipid bilayer occurs primarily through the organic anion-transporting polypeptide 1B1 (OATP1B1) influx transporter which is expressed on the basolateral membrane of human hepatocytes (Figure 2). OATP1B1 transport appears to be rate limiting for hepatocyte uptake and hence distribution and metabolism of many statins. Consequently, modifications in this transporter have been significantly associated with the risk of SRM [26,58,59]. OATP1B1 is encoded by the gene *SLCO1B1* whose *5 allele (Val174Ala, 521T>C) has been shown to interfere with localization of the transporter to the plasma membrane, leading to decreased liver uptake and greater systemic statin concentrations and hence greater muscle statin exposure [60].

The genotypic frequencies for the variants of *SLCO1B1* vary by ethnicity and some reduced function alleles are relatively common, such as the presence of *5 in 8%–20% of Caucasians. The best-documented haplotypes thought to play an important role in modulating the risk of SRM are shown in Table 3. Of note, the *15 haplotype, with an allele frequency of 10% in Japanese, carries the same 521T>C substitution as *5 in combination with the 388A>G SNP and represents another risk allele for myopathy in patients receiving statin therapy.

**Table 3:** *SLCO1B1* Haplotypes in Various Ethnic Groups. Adapted with permission from Oshiro *et al*. [60].

Nucleotide Change (s)	rsID	Protein Variation (s)	Haplotype	Transporter Effect	OATP1B1 substrate serum conc.	Allele Frequency (%) ^a^				Ref.
						AA	J	As	C	
**None**	N/A	N/A	*1A	Normal	Baseline					[60]
**388A>G**	2306283	Asn130Asp	*1B	Increased	Decreased	74–78	54	58–81	37–46	[60]
**521T>C**	4149056	Val174Ala	*5	Reduced	Increased	1–4	0.7	6–19	12–20	[60]
**521T>C+ 388A>G**	4149056+ 2306283	Val174Ala+ Asn130Asp	*15	Reduced	Increased		10			[60]

^a^ AA = African American [61,62], J = Japanese [63], As = Asian [64,65], C = Caucasian [61,62,66,67].

### 4.2. Pharmacokinetic Evidence of a Class-Effect

Reduced function mutations in *SLCO1B1* limit the transport of these molecules by OATP1B1 into liver cells, which leaves an increased concentration of the statin in the bloodstream. Elevated plasma concentrations of statins increase the risk of adverse drug reactions, of which SRM is one of the most common. This effect has been best documented for simvastatin where the Area Under the Concentration-response Curve [68] (**AUC_0-__∞_** (ng∙hr/mL)) is greater than three times higher in those patients homozygous for the *5 variant than wild types after a single 40-mg dose [57]. By contrast, fluvastatin has been shown to have pharmacokinetic properties that are independent of genetic variation at the *SLCO1B1* locus, and notably has correspondingly lower rates of reported SRM [69]. 

Although most statins are substrates of the transporter OATP1B1 [69], the effects of *SLCO1B1* polymorphism still vary based on the pharmacologic profile of the specific statin. Statins each possess unique absorption, distribution, metabolism, and excretion properties that affect kinetics and treatment response (Table 4). 

**Table 4:** Statin Pharmacologic Properties.

	Fluvastatin	Rosuvastatin	Pitavastatin	Pravastatin	Lovastatin	Atorvastatin	Simvastatin
**Elimination Half-Life**	3 h	19 h	11 h	2 h	4h	14 h	4 h
**LDL Lowering Potency**	Low	High	Low-Mod	Low-Mod	Low-Mod	Mod-High	Mod
**Renal excretion**	5%	90%	15%	20%	10%	2%	13%
**OATP1B1 dependence**	-	-	+/-	+	++	+++	++++
**Starting dose ^a^**	80 mg XL HS	5-10 mg QD	1-2 mg QD	20-40 mg HS	20-40 mg HS	10-20 mg QD	20-40 mg HS

^a^ QD = Once Daily, HS = Taken at bedtime, LDL = Low Density Lipoprotein.

### 4.3. Clinical Evidence

If toxicity is correlated with muscle exposure to drug levels, then logically, increased drug concentrations in the blood should be reflected in an altered side effect profile in patients with reduced transport. Several safety studies have evaluated how the rates of adverse drug reactions, most commonly myopathy, vary by genotype. To date, five studies have evaluated the risk of SRM as a function of genetic variation in *SLCO1B1* (Table 5).

**Table 5:** *SLCO1B1* and Risk of Myopathy. S = Simvastatin, A = Atorvastatin, R = Rosuvastatin, P = Pravastatin, C = Cerivastatin, RR = relative risk, OR = Odds Ratio, ULN = Upper Limit of Normal.

Study	Drug	n	Allele(s)	Clinical Endpoint	Outcome
SEARCH [59]	S 80 mg	175	*5	Definite or incipient myopathy	OR = 4.7 per copy (*p* = 3 × 10^−28^)
HPS [59]	S 40 mg	1,664	*5	Definite or incipient myopathy	OR = 2.6 per copy (*p* = 0.004)
STRENGTH [58]	S20→80 mg	452	*5	Composite adverse event (CAE) defined as discontinuation for any side effect, myalgia, or CK>3x ULN	S: OR = 1.7 per copy (*p* = 0.03)
	P10→40 mg				
	A10→80 mg				
GO-DARTS [26]	All Statins, all doses	4,141	*1B, *5, *15	Intolerance as defined by an increase in CK (1xULN>CK<3xULN) or ALT and aberrant prescription patterns	OR = 2.05, (*p* = 0.043)
Marciante *et al*., 2011 [70]	C	917	*5	Rhabdomyolysis	OR = 1.89, (*p* = 0.002)

The myopathy risk associated with *SLCO1B1* was first reported in a study reported by the Study of the Effectiveness of Additional Reductions in Cholesterol and Homocysteine (SEARCH) Collaborative Group [59]. The authors studied two cohorts of clinically severe cases and controls from large trials involving approximately 12,000 and 20,000 participants who were treated with 80 mg and 40 mg of simvastatin per day, respectively. The investigators observed a significant association between SRM and a single marker in the *SLCO1B1* gene (rs4363657, *p* = 3 × 10^−28^). This association was then confirmed in a second cohort which included patients who were randomly assigned to 40 mg of simvastatin per day (see Table 6). 

**Table 6:** Myopathy Risk in SEARCH Stratified by SLCO1B1 Genotype [59].

Genotype ^a^	Population Frequency	Cumulative Percentage with Myopathy	
		Year 1	Year 5
**Wild Type**	73%	0.34%	0.63%
**Heterozygote**	24.9%	1.38%	2.83%
**Homozygote**	2.1%	15.25%	18.55%

^a^ Wild Type-521TT, Heterozygous-521TC, Homozygous-521CC.

The results from the retrospective genetic association study in SEARCH and HPS were subsequently validated in the prospective randomized STRENGTH (Statin Response Examined by Genetic Haplotype Markers) study [58]. In STRENGTH, subjects (n = 509) were randomized to ascending doses of atorvastatin 10→80 mg, simvastatin 20→80 mg, or pravastatin 10→40 mg. A composite adverse event (CAE) was defined as discontinuation for any side effect, myalgia, or CK>3 times upper limit of normal (ULN) during follow-up. Of the five candidate genes evaluated, including *CYP2D6*, *CYP2C8*, *CYP2C9*, *CYP3A4*, and *SLCO1B1*, only SLCO1B1*5 was associated with CAE (37% *vs*. 25% in carriers and wild type patients respectively, *p* = 0.03) and more significantly for those with CAE exclusive of significant CK elevation (*p* ≤ 0.03). Furthermore, a gene-dosage effect was observed (percent with CAE in those with 0, 1, or 2 of the variant (*5) alleles: 19%, 27%, and 50%, *p* = 0.01 for the trend). Importantly, only allele carriers receiving ascending doses of simvastatin showed significantly heightened risk of CAE compared to patients who carried no alleles (16% *vs.* 34%, *p* = 0.01). This is in contrast to patients receiving atorvastatin and pravastatin who showed non-significant changes in CAE risk based on allele carriage (19% *vs.* 27%, *p* = 0.3 and 22% *vs.* 22%, *p* = 0.97 for atorvastatin and pravastatin respectively).Since carriers of 521T>C mutations experienced higher rates of myalgia, a significant obstacle to optimal adherence, these same patients should have prescribing patterns reflective of intolerance such as switching to a different statin at a lower or equivalent dose, reducing the dose of the same statin, or discontinuation of statin therapy. This hypothesis was the aim of the GO-DARTS (Genetics of Diabetes Audit and Research) study which examined whether *SLCO1B1* variants were associated with general statin intolerance in a large population of patients with type 2 diabetes receiving statins as part of routine clinical care. This observational incident cohort analysis used information from 4,196 genotyped patients in the GO-DARTS database, which is part of an ongoing research initiative in the Tayside, Scotland (population 400,000) community to track the treatment and health outcomes of individuals with diabetes [26]. Information captured in this database included detailed clinical information for individuals with diabetes from 1990 to the present including all pharmacy records, lab test results, and other clinical data related to diabetes care. This study particularly focused on mild manifestations of myopathy, and patients with CK > 3×ULN*^iv^* [71] were excluded from analysis. For purposes of this study, intolerance was defined as a composite measure of abnormal lab values, alanine transaminase (ALT) and CK, and relevant adjustments to the prescription of each patient. 

This study confirmed the association of the *5 allele with statin intolerance (OR = 2.05, 95% CI: 1.02–4.09, *p* = 0.04), and further showed that *5 allele carriers have a doubled risk for intolerance to their originally prescribed statin. These results were observed in a population where moderate and severe cases of myopathy were excluded, therefore representing better the sub-pathological end of the spectrum of statin related muscle effects likely to be the more significant driver of correlated non-adherence in terms of numbers. This study suggests that the muscle toxicity associated with *SLCO1B1* is represented in prescribing patterns suggestive of intolerance, and may ultimately prove to be useful as a prospective intervention.

Although the majority of evidence for *SLCO1B1-*related SRM has been around simvastatin, cerivastatin, a drug that was recalled due to its risk of rhabdomyolysis [4], has also recently been shown to be effected by this locus. In an analysis by Marciante *et al*., a candidate gene study (examining *CYP2C8*, *UGT1A1*, *UGT1A3*, and *SLCO1B1*) and a GWAS study were performed on 185 cerivastatin-induced rhabdomyolysis cases matched to statin-using controls from Cardiovascular Health Study (n = 374) and Vascular Health Study (n = 358) [70], A subsequent in vitro functional analysis for 521T>C was also performed in stable HEK293 cells. Permutation test results showed an association between cerivastatin-induced rhabdomyolysis and the *5 allele (OR = 1.89, *p* = 0.002). In functional studies, this variant reduced transport by 40% compared with the reference transporter (*p* < 0.001). This study extends the results of simvastatin-centered trials to cerivastatin and functional studies provide a potential causal association. 

### 4.4. SLCO1B1 as an Adherence Intervention

Clinical evidence shows a strong association between carriage of alleles of *SLCO1B1* and both mild myalgia and clinically severe myopathy [58,59]. Furthermore, *SLCO1B1* induced muscle toxicity has also been associated with lower levels of drug tolerance [26]. Since there is a gradient of effect for variations in this transporter across the statin class [72], it may be possible to personalize statin treatment for the patient’s effectiveness goals as well as their predisposition to myopathy according to *SLCO1B1* genotype. In fact various groups, including the Clinical Pharmacogenomics Implementation Consortium, have drafted specific treatment recommendations that can provide clinicians with a practical starting point for how to implement a patient’s 521T>C status into their treatment [72,73].

Although the evidence from the AKROBATS trial may not fully support the utility of genomic testing in itself in improving patient adherence [27], the concept is compelling and the use of *SLCO1B1* may go one step further by not only affecting a patient’s sense of self-efficacy [32] but also reducing the probability of myopathy, an independent barrier to optimal adherence. Most importantly, decisions based on this intervention could lead to less atherosclerosis and cardiovascular events. Currently there is no direct clinical evidence that the personalized prescribing of statins using a patient’s SLCO1B1*5 status will improve their medication adherence; however, previous analyses suggest that this is a logical conclusion and will be an important hypothesis to evaluate in future analyses.

## 5. Conclusions

Although the benefits of statin therapy and the importance of adherence for maximum efficacy have been demonstrated, adherence rates remain low. Patients do not adhere to their medications for many reasons including low health literacy, cost and side effect profiles. Personalized prescribing in the statin class has the potential to improve both the efficacy and safety of these drugs. Testing of *KIF6*, a potential marker of statin effectiveness, has been suggested as a means to select those patients best suited for intensive treatment. Disclosure of this personalized risk may improve a patient’s sense of self-efficacy and therefore improve the likelihood of adherence. This logic has recently been evaluated in some early research with mixed results. *SLCO1B1* is a marker for statin safety and may be capable of personalizing treatment to a patient’s individual risk of myopathy, an independent barrier to optimal adherence. Given the prevalence of statin usage and the important place of these medications in the treatment of the epidemic of CHD, there could be significant benefit from personalizing statin treatment to promote increased patient adherence. 

## References

[1] NHLBI Fact Book. http://www.nhlbi.nih.gov/about/factpdf.htm.

[2] Roger V.L., Go A.S., Lloyd-Jones D.M., Benjamin E.J., Berry J.D., Borden W.B., Bravata D.M., Dai S., Ford E.S., Fox C.S. (2012). Heart disease and stroke statistics—2012 update: A report from the american heart association. Circulation.

[3] IMS Institute for Healthcare Informatics The use of medicines in the united states: Review of 2011. http://www.imshealth.com/ims/Global/Content/Insights/IMS%20Institute%20for%20Healthcare%20Informatics/IHII_Medicines_in_U.S_Report_2011.pdf.

[4] Staffa J.A., Chang J., Green L. (2002). Cerivastatin and reports of fatal rhabdomyolysis. N. Engl. J. Med..

[5] Egan A., Colman E. (2011). Weighing the benefits of high-dose simvastatin against the risk of myopathy. N. Engl. J. Med..

[6] Andrade S.E., Walker A.M., Gottlieb L.K., Hollenberg N.K., Testa M.A., Saperia G.M., Platt R. (1995). Discontinuation of antihyperlipidemic drugs—Do rates reported in clinical trials reflect rates in primary care settings?. N. Engl. J. Med..

[7] Simons L.A., Levis G., Simons J. (1996). Apparent discontinuation rates in patients prescribed lipid-lowering drugs. Med. J. Aust..

[8] Avorn J., Monette J., Lacour A., Bohn R.L., Monane M., Mogun H., LeLorier J. (1998). Persistence of use of lipid-lowering medications: A cross-national study. JAMA.

[9] Flack J.M., Novikov S.V., Ferrario C.M. (1996). Benefits of adherence to anti-hypertensive drug therapy. Eur. Heart J..

[10] Haynes R.B., McKibbon K.A., Kanani R. (1996). Systematic review of randomised trials of interventions to assist patients to follow prescriptions for medications. Lancet.

[11] Osterberg L., Blaschke T. (2005). Adherence to medication. N. Engl. J. Med..

[12] McCarthy R. (1998). The price you pay for the drug not taken. Bus. Health.

[13] Bosworth H.B., Granger B.B., Mendys P., Brindis R., Burkholder R., Czajkowski S.M., Daniel J.G., Ekman I., Ho M., Johnson M. (2011). Medication adherence: A call for action. Am. Heart J..

[14] Berg J.S., Dischler J., Wagner D.J., Raia J.J., Palmer-Shevlin N. (1993). Medication compliance: A healthcare problem. Ann. Pharmacother..

[15] Levy G., Zamacona M.K., Jusko W.J. (2000). Developing compliance instructions for drug labeling. Clin. Pharmacol. Ther..

[16] McDonnell P.J., Jacobs M.R. (2002). Hospital admissions resulting from preventable adverse drug reactions. Ann. Pharmacother..

[17] Cherry S.B., Benner J.S., Hussein M.A., Tang S.S., Nichol M.B. (2009). The clinical and economic burden of nonadherence with antihypertensive and lipid-lowering therapy in hypertensive patients. Value Health.

[18] Roebuck M.C., Liberman J.N., Gemmill-Toyama M., Brennan T.A. (2011). Medication adherence leads to lower health care use and costs despite increased drug spending. Health Aff. (Millwood).

[19] Jackevicius C.A., Mamdani M., Tu J.V. (2002). Adherence with statin therapy in elderly patients with and without acute coronary syndromes. JAMA.

[20] McNicholl I.R. (2008). Strategies to enhance adherence, reduce costs, and improve patient quality of life. J. Manag. Care Pharm..

[21] Marteau T., Senior V., Humphries S.E., Bobrow M., Cranston T., Crook M.A., Day L., Fernandez M., Horne R., Iversen A. (2004). Psychological impact of genetic testing for familial hypercholesterolemia within a previously aware population: A randomized controlled trial. Am. J. Med. Genet. A.

[22] Grant R.W., Hivert M., Pandiscio J.C., Florez J.C., Nathan D.M., Meigs J.B. (2009). The clinical application of genetic testing in type 2 diabetes: A patient and physician survey. Diabetologia.

[23] Umans-Eckenhausen M.A., Defesche J.C., van Dam M.J., Kastelein J.J. (2003). Long-term compliance with lipid-lowering medication after genetic screening for familial hypercholesterolemia. Arch. Intern. Med..

[24] Narod S.A. (2010). Compliance with tamoxifen in women with breast cancer and a brca1 or brca2 mutation. J. Clin. Oncol..

[25] Heshka J.T., Palleschi C., Howley H., Wilson B., Wells P.S. (2008). A systematic review of perceived risks, psychological and behavioral impacts of genetic testing. Genet. Med..

[26] Donnelly L.A., Doney A.S., Tavendale R., Lang C.C., Pearson E.R., Colhoun H.M., McCarthy M.I., Hattersley A.T., Morris A.D., Palmer C.N. (2011). Common nonsynonymous substitutions in slco1b1 predispose to statin intolerance in routinely treated individuals with type 2 diabetes: A go-darts study. Clin. Pharmacol. Ther..

[27] Charland S.L., Agatep B.C., Epstein R.S., Frueh F.W., Herrera V., Devlin J., Superko H., Stanek E.J. (2012). Patient knowledge of pharmacogenetic information improves adherence to statin therapy: Results of the additional kif6 risk offers better adherence to statins (akrobats) trial. J. Am. Coll. Cardiol..

[28] Asbury C.L. (2005). Kinesin: World’s tiniest biped. Curr. Opin. Cell Biol..

[29] Li Y., Iakoubova O.A., Shiffman D., Devlin J.J., Forrester J.S., Superko H.R. (2010). Kif6 polymorphism as a predictor of risk of coronary events and of clinical event reduction by statin therapy. Am. J. Cardiol..

[30] Ference B.A., Yoo W., Flack J.M., Clarke M. (2011). A common kif6 polymorphism increases vulnerability to low-density lipoprotein cholesterol: Two meta-analyses and a meta-regression analysis. PLoS One.

[B31-jpm-02-00158] 31.Self-efficacy in the context of health psychology is defined as a patient’s sense of their capability to improve their health outcomes via behavior change.

[32] Bloss C.S., Madlensky L., Schork N.J., Topol E.J. (2011). Genomic information as a behavioral health intervention: Can it work?. Pers. Med..

[33] Morrison A.C., Bare L.A., Chambless L.E., Ellis S.G., Malloy M., Kane J.P., Pankow J.S., Devlin J.J., Willerson J.T., Boerwinkle E. (2007). Prediction of coronary heart disease risk using a genetic risk score: The atherosclerosis risk in communities study. Am. J. Epidemiol..

[34] Bare L.A., Morrison A.C., Rowland C.M., Shiffman D., Luke M.M., Iakoubova O.A., Kane J.P., Malloy M.J., Ellis S.G., Pankow J.S. (2007). Five common gene variants identify elevated genetic risk for coronary heart disease. Genet. Med..

[35] Shiffman D., Sabatine M.S., Louie J.Z., Kirchgessner T.G., Iakoubova O.A., Campos H., Devlin J.J., Sacks F.M. (2010). Effect of pravastatin therapy on coronary events in carriers of the kif6 719arg allele from the cholesterol and recurrent events trial. Am. J. Cardiol..

[36] Iakoubova O.A., Robertson M., Tong C.H., Rowland C.M., Catanese J.J., Blauw G.J., Jukema J.W., Murphy M.B., Devlin J.J., Ford I. (2010). Kif6 trp719arg polymorphism and the effect of statin therapy in elderly patients: Results from the prosper study. Eur. J. Cardiovasc. Prev. Rehabil..

[37] Iakoubova O.A., Sabatine M.S., Rowland C.M., Tong C.H., Catanese J.J., Ranade K., Simonsen K.L., Kirchgessner T.G., Cannon C.P., Devlin J.J. (2008). Polymorphism in kif6 gene and benefit from statins after acute coronary syndromes: Results from the prove it-timi 22 study. J. Am. Coll. Cardiol..

[38] Shiffman D., Chasman D.I., Zee R.Y., Iakoubova O.A., Louie J.Z., Devlin J.J., Ridker P.M. (2008). A kinesin family member 6 variant is associated with coronary heart disease in the women’s health study. J. Am. Coll. Cardiol..

[39] Iakoubova O.A., Tong C.H., Rowland C.M., Kirchgessner T.G., Young B.A., Arellano A.R., Shiffman D., Sabatine M.S., Campos H., Packard C.J. (2008). Association of the trp719arg polymorphism in kinesin-like protein 6 with myocardial infarction and coronary heart disease in 2 prospective trials: The care and woscops trials. J. Am. Coll. Cardiol..

[40] Allingham-Hawkins D., Lea A., Levine S. (2010). Kif6 p.Trp719arg testing to assess risk of coronary artery disease and/or statin response. PLoS Curr..

[41] Topol E.J., Damani S.B. (2010). The kif6 collapse. J. Am. Coll. Cardiol..

[42] Hopewell J.C., Parish S., Clarke R., Armitage J., Bowman L., Hager J., Lathrop M., Collins R. (2011). No impact of kif6 genotype on vascular risk and statin response among 18,348 randomized patients in the heart protection study. J. Am. Coll. Cardiol..

[43] Ridker P.M., MacFadyen J.G., Glynn R.J., Chasman D.I. (2011). Kinesin-like protein 6 (kif6) polymorphism and the efficacy of rosuvastatin in primary prevention. Circ. Cardiovasc. Genet..

[44] Arsenault B.J., Boekholdt S.M., Hovingh G.K., Hyde C.L., DeMicco D.A., Chatterjee A., Barter P., Deedwania P., Waters D.D., LaRosa J.C. (2012). The 719arg variant of kif6 and cardiovascular outcomes in statin-treated, stable coronary patients of the treating to new targets and incremental decrease in end points through aggressive lipid-lowering prospective studies. Circ. Cardiovasc. Genet..

[45] Assimes T.L., Holm H., Kathiresan S. (2010). Lack of association between the trp719arg polymorphism in kinesin-like protein-6 and coronary artery disease in 19 case-control studies. J. Am. Coll. Cardiol..

[B46-jpm-02-00158] 46.Proportion of days covered (PDC) is a measurement used to quantify a patient’s level of medication adherence. It is defined as a ratio of the number of days of medication that a patient had available to them over the complete coverage period. For example if a patient was given a 60 day statin prescription but only filled their script for one of those months, they would have a PDC of 0.5.

[47] Ghatak A., Faheem O., Thompson P.D. (2010). The genetics of statin-induced myopathy. Atherosclerosis.

[48] US Food and Drug Administration FDA drug safety communication: Ongoing safety review of high-dose zocor (simvastatin) and increased risk of muscle injury. http://www.fda.gov/Drugs/DrugSafety/PostmarketDrugSafetyInformationforPatientsandProviders/ucm204882.htm.

[49] Josan K., Majumdar S.R., McAlister F.A. (2008). The efficacy and safety of intensive statin therapy: A meta-analysis of randomized trials. CMAJ.

[50] Cohen J.D., Brinton E.A., Ito M.K., Jacobson T.A. (2012). Understanding statin use in america and gaps in patient education (usage): An internet-based survey of 10,138 current and former statin users. J. Clin. Lipidol..

[51] Bruckert E., Hayem G., Dejager S., Yau C., Begaud B. (2005). Mild to moderate muscular symptoms with high-dosage statin therapy in hyperlipidemic patients—The primo study. Cardiovasc. Drugs Ther..

[52] Kiortsis D.N., Giral P., Bruckert E., Turpin G. (2000). Factors associated with low compliance with lipid-lowering drugs in hyperlipidemic patients. J. Clin. Pharm. Ther..

[53] Bruckert E., Simonetta C., Giral P. (1999). Compliance with fluvastatin treatment characterization of the noncompliant population within a population of 3,845 patients with hyperlipidemia. Creole study team. J. Clin. Epidemiol..

[54] Mann D.M., Woodward M., Muntner P., Falzon L., Kronish I. (2010). Predictors of nonadherence to statins: A systematic review and meta-analysis. Ann. Pharmacother..

[55] Mann D.M., Allegrante J.P., Natarajan S., Halm E.A., Charlson M. (2007). Predictors of adherence to statins for primary prevention. Cardiovasc. Drugs Ther..

[56] Brown M.T., Bussell J.K. (2011). Medication adherence: Who cares?. Mayo Clin. Proc..

[57] Pasanen M.K., Neuvonen M., Neuvonen P.J., Niemi M. (2006). Slco1b1 polymorphism markedly affects the pharmacokinetics of simvastatin acid. Pharmacogenet. Genomics.

[58] Voora D., Shah S.H., Spasojevic I., Ali S., Reed C.R., Salisbury B.A., Ginsburg G.S. (2009). The slco1b1*5 genetic variant is associated with statin-induced side effects. J. Am. Coll. Cardiol..

[59] Link E., Parish S., Armitage J., Bowman L., Heath S., Matsuda F., Gut I., Lathrop M., Collins R. (2008). Slco1b1 variants and statin-induced myopathy—A genomewide study. N. Engl. J. Med..

[60] Oshiro C., Mangravite L., Klein T., Altman R. (2010). Pharmgkb very important pharmacogene: Slco1b1. Pharmacogenet. Genomics.

[61] Tirona R.G., Leake B.F., Merino G., Kim R.B. (2001). Polymorphisms in oatp-c: Identification of multiple allelic variants associated with altered transport activity among european- and african-americans. J. Biol. Chem..

[62] Mwinyi J., Kopke K., Schaefer M., Roots I., Gerloff T. (2008). Comparison of slco1b1 sequence variability among german, turkish, and african populations. Eur. J. Clin. Pharmacol..

[63] Nozawa T., Nakajima M., Tamai I., Noda K., Nezu J., Sai Y., Tsuji A., Yokoi T. (2002). Genetic polymorphisms of human organic anion transporters oatp-c (slc21a6) and oatp-b (slc21a9): Allele frequencies in the Japanese population and functional analysis. J. Pharmacol. Exp. Ther..

[64] Nishizato Y., Ieiri I., Suzuki H., Kimura M., Kawabata K., Hirota T., Takane H., Irie S., Kusuhara H., Urasaki Y. (2003). Polymorphisms of oatp-c (slc21a6) and oat3 (slc22a8) genes: Consequences for pravastatin pharmacokinetics. Clin. Pharmacol. Ther..

[65] Ho W.F., Koo S.H., Yee J.Y., Lee E.J. (2008). Genetic variations of the slco1b1 gene in the Chinese, Malay and Indian populations of Singapore. Drug Metab. Pharmacokinet..

[66] Couvert P., Giral P., Dejager S., Gu J., Huby T., Chapman M.J., Bruckert E., Carrie A. (2008). Association between a frequent allele of the gene encoding oatp1b1 and enhanced ldl-lowering response to fluvastatin therapy. Pharmacogenomics.

[67] Pasanen M.K., Backman J.T., Neuvonen P.J., Niemi M. (2006). Frequencies of single nucleotide polymorphisms and haplotypes of organic anion transporting polypeptide 1b1 slco1b1 gene in a finnish population. Eur. J. Clin. Pharmacol..

[B68-jpm-02-00158] 68.Area under the curve (AUC) is measure commonly used in pharmacokinetics and is defined as the area under the plot of plasma concentration of drug against time after the initial drug administration. AUC may be used as a proxy measure of systemic exposure to a drug over a given time window.

[69] Neuvonen P.J., Niemi M., Backman J.T. (2006). Drug interactions with lipid-lowering drugs: Mechanisms and clinical relevance. Clin. Pharmacol. Ther..

[70] Marciante K.D., Durda J.P., Heckbert S.R., Lumley T., Rice K., McKnight B., Totah R.A., Tamraz B., Kroetz D.L., Fukushima H. (2011). Cerivastatin, genetic variants, and the risk of rhabdomyolysis. Pharmacogenet. Genomics.

[B71-jpm-02-00158] 71.Upper limit of normal (ULN) is the upper threshold value of a normal range for a defined laboratory measure. >3×ULN would be a value that is greater than threefold higher than the upper limit of a normal range.

[72] Niemi M. (2010). Transporter pharmacogenetics and statin toxicity. Clin. Pharmacol. Ther..

[73] Wilke R.A., Ramsey L.B., Johnson S.G., Maxwell W.D., McLeod H.L., Voora D., Krauss R.M., Roden D.M., Feng Q., Cooper-DeHoff R.M. (2012). The clinical pharmacogenomics implementationconsortium: Cpic guideline for slco1b1 and simvastatin-induced myopathy. Clin. Pharmacol. Ther..

